# Estimating the Risk of Maternal Death at Admission: A Predictive Model from a 5-Year Case Reference Study in Northern Uganda

**DOI:** 10.1155/2022/4419722

**Published:** 2022-03-17

**Authors:** Gasthony Alobo, Cristina Reverzani, Laura Sarno, Barbara Giordani, Luigi Greco

**Affiliations:** ^1^Department of Obstetrics and Gynaecology, Lira University, Lira, Uganda; ^2^Department of Obstetrics and Gynaecology, St. Mary's Hospital Lacor, Gulu, Uganda; ^3^Department of Neurosciences, Reproductive Science and Dentistry, University of Naples Federico II, Naples, Italy; ^4^GULUNAP Project, Department of Paediatrics, University of Naples Federico II, Naples, Italy

## Abstract

**Background:**

Uganda is one of the countries in the Sub-Saharan Africa with a very high maternal mortality ratio estimated at 336 deaths per 100,000 live births. We aimed at exploring the main factors affecting maternal death and designing a predictive model for estimation of the risk of dying at admission at a major referral hospital in northern Uganda.

**Methods:**

This was a retrospective matched case-control study, carried out at Lacor Hospital in northern Uganda, including 130 cases and 336 controls, from January 2015 to December 2019. Multivariate logistic regression was used to estimate the net effect of the associated factors. A cumulative risk score for each woman based on the unstandardised canonical coefficients was obtained by the discriminant equation.

**Results:**

The average maternal mortality ratio was 328 per 100,000 live births. Direct obstetric causes contributed to 73.8% of maternal deaths; the most common were haemorrhage (42.7%), sepsis (24.0%), hypertensive disorders (18.7%) and complications of abortion (2.1%), whereas malaria (23.5%) and HIV/AIDS (20.6%) were the leading indirect causes. The odds of dying were higher among women who were aged 30 years or more (OR 1.12; 95% CI, 1.04–1.19), did not attend antenatal care (OR 3.11; 95% CI, 1.36–7.09), were HIV positive (OR 3.13; 95% CI, 1.41–6.95), had a caesarean delivery (OR 2.22; 95% CI 1.13–4.37), and were referred from other facilities (OR 5.57; 95% CI 2.83–10.99).

**Conclusion:**

Mortality is high among mothers referred late from other facilities who are HIV positive, aged more than 30 years, lack antenatal care attendance, and are delivered by caesarean section. This calls for prompt and better assessment of referred mothers and specific attention to antibiotic therapy before and after caesarean section, especially among HIV-positive women.

## 1. Background

Worldwide, about 830 women die from pregnancy and childbirth-related complications every day [1]. It has been reported that in 2015, about 303,000 women died during and following pregnancy and childbirth, and most of these deaths could have been prevented [1]. The high number of maternal deaths in some parts of the world reflects inequities in access to health services and highlights the gap between the rich and the poor [2]. Almost all maternal deaths (99%) occur in low- and middle-income countries (LMICs) [3]. More than half of these deaths occur in Sub-Saharan Africa (SSA) and about a third in South Asia [2, 4]. These are usually fragile, humanitarian, and postconflict areas [5]. Despite the decline in the global trend of maternal mortality ratio (MMR), estimated at 5.4% between 1990 and 2015, this is not the case for SSA where the decline is still very low and has remained stagnant in some areas [1, 4, 6].

Also, the burden of maternal death is quite significant; most women who die lose between 39.8 and 41.5 years of life [7]. Many women also get sequelae after a near miss event; it has been estimated that for every maternal death about 6 women suffer severe morbidities, some of which occur lifelong [8, 9]. Because of the high burden of maternal morbidity and mortality, ending preventable maternal mortality is a priority under the Sustainable Development Goals (SDG) agenda. In fact, SDG target 3.1 aims to reduce the average global MMR to less than 70 maternal deaths per 100,000 live births by 2030 [10].

A key requirement for further advances in the reduction of maternal deaths through effective policy and health program decisions is to understand the causes of deaths. By definition, a maternal death refers to the death of a woman during pregnancy or within 42 days of delivery or termination of pregnancy, due to any cause related to, or aggravated by the pregnancy or its management, but excluding deaths from incidental or accidental causes [11]. This allows the identification of maternal deaths based on their causes, as either direct or indirect [12]. In a large World Health Organization (WHO) systematic analysis, 73% of all maternal deaths worldwide were due to direct causes, and 23% to indirect causes. Among the direct causes, haemorrhage accounted for 27.1%, hypertensive disorders 14.0%, sepsis 10.7%, abortion 7.9%, and others 12.8% [13]. Indirect causes varied greatly, but the overall proportion of HIV deaths was highest in SSA [13]. However, the causes of maternal deaths are usually influenced by some non-medical factors, which lead to a delay in accessing maternal healthcare services. The “three delays” model has been widely used to understand the factors that contribute to maternal death. The first delay is the delay to decide to seek care, the second delay is the delay to reach the health facility, and the third delay is the delay to receive care at the health facility [14].

In Uganda, slow progress has been made in reducing maternal deaths, despite the relevant increase in skilled birth attendance from 59% [15] to 73% [16] and antenatal care attendance from 95% to 97% between 2011 and 2016. The maternal mortality ratio only declined from 438 deaths [15] to 336 deaths per 100,000 live births [15]. Most of the women who die are from rural and hard-to-reach areas, and they are usually uneducated and HIV positive; they generally delay seeking care and lack male partner support [17]. The leading causes of maternal death are direct obstetric causes and include haemorrhage, infection, hypertensive disorders, and abortion complications [8, 16]. HIV/AIDS also contributes significantly to indirect maternal deaths at some tertiary hospitals in Uganda [18].

Uganda is one of the countries with a very high maternal mortality ratio in Sub-Saharan Africa, estimated at 336 deaths per 100,000 live births [16]. It is recommended by the Ministry of Health (MoH) that all maternal deaths are reviewed, following the development of maternal death review (MDR) guidelines in 2004, based on WHO guidance “Beyond the numbers” [19]. MDR helps to identify the direct and indirect causes of maternal death and the underlying factors. Consequently, the potentially avoidable factors, missed opportunities, and substandard care are identified, and actionable recommendations are made to avert a reoccurrence of deaths under similar circumstances.

Although maternal death reviews are done in most of the facilities in northern Uganda, and in particular, at Lacor Hospital, a significant number of women continue to die due to pregnancy and delivery-related complications. Very few studies have been carried out to determine the factors that contribute to these delays in central and western Uganda. However, no studies are available for northern Uganda. Therefore, this study aimed at exploring the main factors affecting maternal deaths in northern Uganda, after a 20-year-old conflict, and designing a predictive model for estimation of the risk at admission of the mothers in the hospital.

## 2. Methods

### 2.1. Study Design, Study Population, and Setting

This was a retrospective matched case-control study with cases to controls ratio of 1 : 3. All consecutive cases of maternal deaths reported at the Maternity Unit of St. Mary's Hospital Lacor over the years 2015–2019 were collected. For each year, every case was matched with 3 controls for the district of residence.

These cases were maternal deaths, which were regularly reviewed and notified to the Ministry of Health (MoH) from January 2015 to December 2019.

Controls were women admitted to the maternity ward at Lacor Hospital, either in labour or with pregnancy-related complications like medical illnesses in pregnancy, abortions, and ectopic pregnancy, and discharged home alive after management in the hospital for the same period.

Cases with missing records and women who died before arrival at the hospital (approximately 3%) were not included in this study.

This study was carried out at St. Mary's hospital, Lacor, herein referred to as Lacor Hospital. The hospital is located in Gulu district, in the northern part of Uganda, which is still recovering from a 20-year-old conflict that left many people displaced from their homes and economically disadvantaged. According to Maternal Child Health (MCH) indicators, the situation in Northern Uganda is even worse than that of the rest of the country [16]. Lacor Hospital is a private, nonprofit hospital that serves the biggest population in this region as a general hospital, with specific priority to MCH. It has a 450-bed capacity and on average 20 deliveries per day, reaching the highest number of deliveries in the region. It also records the highest number of referrals from other health centres and unfortunately, also the highest number of maternal deaths in the region.

### 2.2. Sample Size Calculation

Dichotomous endpoint, two independent study sample size was computed. Using data from our setting, the anticipated incidence of mortality is estimated at 10% in the control group and 20% in the case group. Aiming at a first degree error of 0.05 and power of 80%, with a control to case ratio of 3 : 1, we would have required a total sample of 377 controls and 124 cases. Our sample size of 496 (130 cases, 366 controls) was adequate to get 80% power in the study [20, 21].

### 2.3. Data Collection and Entry

Data were collected by reviewing all the medical records and post mortem reports, if available, of the cases and the controls in the period of January 2015 to December 2019. The tool for data collection was modified from the Health Management Information System form 120b (maternal death review form) of the Ugandan MoH. For each included patient, we recorded age, gravidity, parity, referral status, gestational age, marital status, antenatal care (ANC) attendance, HIV serostatus, mode of delivery, duration of stay in the hospital, and cause of death. The lead investigator and another independent doctor reviewed 30% of the data entry forms for completeness and ensured quality control.

### 2.4. Statistical Analysis

Variables were explored for their distribution and appropriate parametric/nonparametric tests were applied. Continuous variables were reported as median (interquartile range-IQR), while categorical variables were reported as number (percentage). Differences between the means of continuous variables were estimated by the ANOVA model. The chi-square test was used to compare percentages. Because most variables were correlated, we explored, using a multivariate model, the power to discriminate between the group of cases and that of controls.

Setting the outcome (alive or dead) as the dependent variable, the odds associated with each risk factor were examined by a multivariate logistic model.

Variables that showed differences between cases and controls in a univariate fashion were added to a multivariate discriminant model to evaluate their contribution to differentiate maternal deaths from survivors. Wilks' lambda estimates the capacity of the model to distinguish between the two groups, where 1 = complete overlap and 0 = complete distance. The variance ratio F estimates the contribution of each variable to the model. The first variable best to discriminate is added to the model, starting from Wilk's lambda of 1 (complete overlap between the two groups) and once that is considered, all others significantly contributing to lowering the Wilks' lambda (and increasing the distance between the two groups) were added.

The model was corrected for possible biases using the auto-exclusion jack-knife method. A logistic regression analysis was adopted to estimate the odds ratio associated with each risk factor.

### 2.5. The Predictive Model for the Risk of Dying at Admission

Women at higher risk of dying may be identified at admission to the hospital by calculating a Discriminant Score (D-score). In this study, the D-score was obtained by multiplying the Canonical Unstandardised Discriminant Coefficient by the corresponding risk value (i.e. 1, if the risk is absent, or 2, if the risk is present); by adding the obtained products; and finally, by adding the constant −6.344.

### 2.6. Ethics Consideration

The Lacor Hospital Institutional Research Ethics Committee gave ethical approval for this study and also granted a waiver of consent since this study did not involve interaction with participants. We obtained permission from the Lacor Hospital authority to retrieve and review the patients' charts. Confidentiality was maintained throughout the study.

## 3. Results

In this matched case-control study, we analysed a total of 496 charts. These included 130 cases (26.2%) and 366 controls (73.8%). Concerning the maternal characteristics, cases were significantly older, had a higher parity, and a lower gestational age at admission than controls as summarised in Table 1.

As concerns the analysis of patients that reached Lacor Hospital as referrals from other health facilities vs. the patients that were directly admitted to the hospital, the former group showed a significantly higher number of emergency operations (69 out of 141 cases), corresponding to 48.9% of the referred patients, while only 25.3% (68 out of 269) of the nonreferred patients delivered by C/S.

The majority of the cases were women referred to Lacor Hospital from various health centres and local hospitals (87/130, 66.9%), while only (93/366, 25.4%) of the control women were referred (Chi Sq = 72, *p* < 0.00001).

Attendance at ANC was remarkably higher in the control group (277/364, 76.1%) than in the case women (72/129, 55.8%; Chi Sq = 18, *p* < 0.0001).

Also, HIV serostatus proved to be a significant risk factor for maternal mortality, with a significantly higher presence of HIV-positive women in the case group (20/74.27.0%) vs. the control group (31/294,10.5%; Chi Sq = 13.4, *p* < 0.001).

The analysis of the type of delivery, i.e., vaginal delivery versus C/S (both elective and emergency) showed higher mortality associated with a caesarean section; in fact, while the women of the control group had a prevalence of vaginal delivery (226/311, 72.7%), the case group had a majority of caesarean section (49/99, 49.5%; Chi Sq = 21.4, *p* < 0.0001).

The marital status did not seem to play a role in the outcome of the delivery, since both the control and case groups evidenced a similar preponderance of married women, of 83.6% and 83.1%, respectively.

In a limited number of cases (*N* = 67), one or more social factors contributed to the maternal death; in particular, there was a delay in the woman seeking help in 58 cases (86.6%), a lack of male support in 2 cases (3.0%), and a lack of transport from home to the health facility in 2 cases (3.0%).

Finally, as concerns health system factors that may have contributed to the women's death, inappropriate intervention played the major role (*N* = 21, 31.3%), followed by a delayed referral (*N* = 17, 25.4%) from a lower health centre to the hospital and the lack of blood and drugs (*N* = 16, 23.9%).

The trend of MMR at Lacor Hospital in the analysed period was unstable, with a generalised improving tendency; after a reduction of the MMR from 298 maternal deaths per 100,000 live births in 2015, to 240 in 2016, the MMR showed then shot to 489 in 2017 and was then followed by more encouraging results in 2018 and 2019 of 324 and 288, respectively.

The causes of maternal deaths were predominantly direct (73.8%, *N* = 96), while the indirect causes of death amounted to 26.2% (*N* = 34).

Concerning the direct maternal deaths, haemorrhage and sepsis were the leading causes thereof, contributing to 31.6% (*N* = 41) and 17.7% (*N* = 23) respectively; 11 cases of haemorrhage (26.8%) were due to ruptured uterus. Other major direct causes of maternal deaths were hypertensive disorders in pregnancy (13.8%, *N* = 18) and abortion (1.5%, *N* = 2).

Among the indirect causes of death, malaria, HIV, cardiovascular diseases, and liver diseases contributed to 6.2% (*N* = 8), 5.4% (*N* = 7), 3.8% (*N* = 5), and 3.8% (*N* = 5), respectively. Less common causes were malignancy (2.4%, *N* = 3) and minor causes classified as “others” (4.6%, *N* = 6).

Table 2 analyses the age, parity, gestational age, and length of stay in the hospital of the maternal deaths determined by direct causes of death. The patients who died of uterine rupture showed a higher age vs. the ones that died of other causes of haemorrhage and sepsis, respectively, of median 32 (95% CI, 29–38) vs. 29 (95% CI, 25–32) and 26.5 (95% CI, 16–36) years of age (*p*=0.019), and a higher parity, respectively, median 5 (95% CI, 4.2–6.8) vs. 3 (95% CI, 2–5) and 1.5 (95% CI, 1–6) (*p*=0.098).

The median gestational age was also different among the direct causes of death but did not reach statistical significance (*p*=0.55). The length of stay in the hospital before the death was median 1 (95% CI, 1–7) day in case of uterine rupture vs. 2 (95% CI, 1.7–4) days in case of other causes of haemorrhage vs. 7.5 (95% CI, 1–22) in the case of sepsis (*p*=0.0001).

Among the direct causes of deaths of the 82 patients that delivered at Lacor Hospital, haemorrhage was the leading cause for women who had both vaginal delivery (52.8%) and C/S (40.9%), as shown in Table 3; the occurrence of sepsis was higher among women who delivered by C/S (29.5%) vs. vaginal delivery (19.4%).

Results of the logistic regression analysis and the discriminant analysis are reported in Table 4. Cases had multiple risks associated to Age >30 years (+110% risk), lack of antenatal care (+310% risk), HIV-positive serostatus (+310% risk), surgical delivery (+220% risk), and being referred (+560% risk). As shown in Table 4, the variable ‘referred (yes)' is the best to differentiate the two groups, followed by age (>30 years), antenatal care (no attendance), positive HIV serostatus, and surgical delivery.

By the discriminant equation produced by the model, we could try to predict which individual is more likely to be classified in the group of cases or the group of controls.

**Figure 1:**
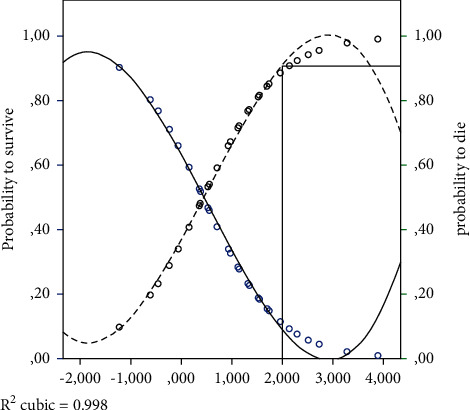
Probability of surviving, (a) or dying, (b) by the Discriminant Score.

For each individual, by multiplying the value of each risk factor for the unstandardised canonical discriminant coefficient (in a linear combination), the probability to be assigned in one of the groups is obtained, ignoring the origin of the case (Table 5). Among 260 controls, 200 are correctly classified as alive by equation (77%) and 60 are wrongly classified as dead (23%), while among 57 cases, 47 are correctly predicted as dead (82.5%), while 10 are wrongly predicted as alive. By plotting the obtained D-score on the probability graph shown in Figure 1, the probability of that woman to survive or to die was estimated. Obviously, there was a large overlapping area (around a D-score value from 0 to 1) over which it was unsafe to make a prediction, but all women with a D-score above 1 have a 70% probability to die, and a woman with a D-score above 2 had a 90+% probability to die (Figure 1). Overall, the equation allowed a correct prediction of the outcome in 78% of women.

Since the prediction shown above is based on an equation derived by the same women who are then classified, the classification results might be over-enthusiastic. Then a jack-knifing procedure was adopted to eliminate this possible bias. The equation was iteratively calculated on all women but one, who was excluded from the equation but was then classified by the equation to which she did not contribute. This was repeated for all women. The actual results were not different from those discussed above: 77% of women were correctly predicted by the discriminant equation.

## 4. Discussion

### 4.1. Direct and Indirect Causes of Maternal Death

In this study, more women died of direct obstetric causes than indirect causes. The leading causes of maternal death were haemorrhage followed by sepsis, hypertensive disorders, and complications of abortion. This finding is similar to those from studies done in LMICs and developed countries [22–24]. In contrast, a study carried out at a teaching hospital in the western part of Uganda found that puerperal sepsis was the leading cause of maternal death followed by haemorrhage at 30.1% and 21.6%, respectively [17]. However, they attributed this result to a lack of preoperative antibiotics.

Our findings show that the risk of dying from obstetric haemorrhage was higher among women who had a vaginal delivery, while women who delivered by caesarean section were at a higher risk of dying due to puerperal sepsis. Except for other confounding factors, some studies found that women who deliver vaginally usually develop postpartum haemorrhage due to uterine atony or genital laceration, whereas those who undergo emergency caesarean section get puerperal sepsis due to inadequate antibiotic therapy [17, 25–27]. Additionally, women who die from haemorrhage spend less time in the hospital than those who die from sepsis because they die more quickly, similar to findings from other studies [27, 28]. Hence, management of obstetric haemorrhage should be done swiftly.

In our study, malaria and HIV were the leading indirect causes of maternal death, similar to findings in other regions of Uganda and the SSA where there is a high prevalence of malaria and HIV [17, 23, 29]. Our findings also show that cardiovascular diseases, mainly rheumatic heart disease and peripartum cardiomyopathy contributed significantly to indirect maternal death, similar to an autopsy-based study that was carried out in Nigeria [30].

### 4.2. Women Most Likely to die

In most resource-limited large maternities, obstetric emergencies present quite a complex and challenging situation right from the point of admission [13, 31]. Hence, it appears more appropriate to propose the identification at admission of women at risk of dying while giving birth. These women could have a special emergency track to save their lives. In the predictive model, we analysed risk factors and the results of the discriminant analysis to compute a cumulative risk score for each woman on admission based on the unstandardised canonical coefficients obtained by the discriminant equation. Our findings suggest that women over 30 years of age are at a higher risk of dying. This was in contrast to most studies done in developed countries and LMICs, where maternal mortality is highest in the younger age groups below 25 years [24, 32, 33]. However, in our study, the age group over 30 years was also associated with poor ANC attendance. Also, the grand multiparous women were at an increased risk of dying. Moreover, on multivariate analysis, high parity was associated with low ANC attendance. Both high parity and low ANC attendance have been associated with poor knowledge of danger signs in pregnancy and a lack of screening for risk factors, all of which increase the risk of maternal death [24]. Furthermore, findings from our study indicate that women living with HIV are at a higher risk of dying compared to those who are HIV negative. HIV/AIDS is strongly linked to maternal mortality even in countries with good antiretroviral treatment in pregnancy [18, 34].

In this present study, we found that women who underwent surgical delivery like caesarean section and exploratory laparotomy had an increased risk of dying; this could be confounded by the indications of the operation. Indeed, in general, surgical deliveries are performed in case a complication occurs. Also, other studies done in LMICs found a positive correlation between operative delivery and maternal death and morbidity [35, 36]. Whereas our study did not evaluate the referral system, evidence from this study showed that women who were referred from other health centres and hospitals were at a higher risk of dying than nonreferred patients. Being referred from a lower facility is linked to late presentation and critical condition at admission [13]. In another retrospective study that was done in Indonesia, it was found that women who were referred from lower health centres had better outcomes than nonreferred cases. They attributed this finding to the timely referral that is being encouraged to avert poor maternal and foetal outcomes [37].

Obviously, the attribution of risk is effective only in about two-thirds of the women in our study. Others die when they do not have any clear-cut risk at admissions, such as primiparae for sepsis or young women after caesarean section. However, our model gives us the possibility to identify high-risk cases that could have a special emergency track to save their lives right from the point of triage ad admission.

### 4.3. Limitations of the Study

There are some limitations to this study that warrant caution when generalising findings. We relied entirely on data contained in the patients' charts, and therefore, some sociodemographic factors that were not captured at admission could not be ascertained. For example, we did not collect data on the level of education and distance from the hospital. We acknowledge that some of these factors may impact maternal mortality as well. Nevertheless, the concept and understanding used in this study can be transferable, and this study brings out some glaring risks of maternal death in a postconflict and rural setting.

## 5. Conclusion

Although maternal mortality remains high at Lacor Hospital with an MMR almost the same as the national average, it is possible to predict the risk of dying from various causes and hence develop ways of reducing it. Most deaths result from direct obstetric causes with haemorrhage, sepsis, and hypertensive disorders leading. Among the indirect causes, malaria and HIV/AIDS are the leading causes of maternal mortality. These are usually women who are referred from other health centres late and, in most cases, have high parity and little or no ANC attendance.

This study, therefore, calls for better assessment and prompt management of referred patients right from the point of admission because most of them die within 24 hours of admission. Additionally, specific attention should be paid to prophylaxis and treatment of infection after caesarean section, as women in this group were more likely to die from puerperal sepsis.

## Figures and Tables

**Table 1:** Maternal characteristics of cases and controls.

Maternal variable	Cases, *n* = 130	Controls, *n* = 366	*P* value
Maternal age (years)	27.85 (SD 6.71)	24.54 (SD 6.42)	<0.0001
Parity	3.62 (SD 2.43)	2.70 (SD 2.06)	<0.0001
Gestational age (weeks)	31.4 (SD 7.94)	37.6 (SD 4.01)	<0.0001
Length of hospitalisation (days)	5.43 (SD 8.10)	3.25 (SD 3.43)	<0.0001
Marital status- married	108 (83.1%)	306 (83.6%)	<0.0001
Referred	87 (66.9%)	93 (25.4%)	<0.00001
ANC attendance	72 (55.8%)	277 (76.1%)	<0.0001
HIV positive	20 (27.0%)	31 (10.5%)	<0.001
Mode of delivery: vaginal delivery, C/S, operative vaginal delivery	47 (47.5%), 49 (49.5%), 2 (2.0%)	226 (72.7%), 73 (23.5%), 1 (0.3%)	<0.0001

**Table 2:** Age, parity, gestational age, and length of stay in the hospital by direct cause of death.

Direct cause of death		Age (years)	Parity	Gestational age (weeks)	Length of stay in the hospital (days)
Haemorrhage (excluding uterine rupture)	Median	29	3	38	2
	Number	30	29	19	30
	95% CI	25–32	2–5	30–38	1.7–4
Uterine rupture	Median	32	5	39	1
	Number	11	10	8	11
	95% CI	29–38	4.2–6.8	31–42	1–7
Sepsis	Median	26.5	1.5	34	7.5
	Number	23	22	7	23
	95% CI	16–36	1–6	15–42	1–22
Hypertensive disorders	Median	28	2	32	2
	Number	18	17	14	18
	95% CI	24–32	2–3.7	28–34	1–7.6
Others	Median	34	3.5	36	1
	Number	14	12	7	14
	95% CI	23–39	1–8	24–43	1–3

**Table 3:** Direct cause of death and type of delivery.

Type of delivery	Direct cause of death					
	Haemorrhage	Sepsis	Hypertensive disorders	Abortion	Others	Total
Vaginal delivery	19 (52.8%)	7 (19.4%)	7 (19.4%)	1 (2.8%)	2 (5.6%)	36 (100,0%)
C/S	18 (40.9%)	13 (29.5%)	7 (15.9%)	0	6 (13.6%)	44 (100.0%)
Vacuum extraction	0	0	1 (50.0%)	0	1 (50.0%)	2 (100.0%)
Total	37	20	15	1	9	82
	45.1%	24.4%	18.3%	1.2%	11.0%	100.0%

**Table 4:** Logistic regression analysis and discriminant analysis.

Variable	Risk	Case	CTRL	Or (CI)	Wilks' lambda	Variance ratio F	*P* value
Age	>30 years	56.1	24.9	1.11 (1.03–1.19)	0.835	30.627	<0.001
ANC	Not attended	44.2	24.0	3.10 (1.36–7.09)	0.809	24.467	<0.001
HIV serostatus	Positive	27.0	10.5	3.12 (1.40–6.94)	0.789	20.668	<0.001
Mode of delivery	Surgical	52.5	27.3	2.22 (1.13–4.36)	0.780	17.408	<0.001
Referral status	Referred	67.4	25.5	5.57 (2.82–10.98)	0.891	38.312	<0.001

**Table 5:** Variables, risk values, and Canonical Unstandardised Discriminant Coefficients.

Variable	Risk value	Canonical Unstandardised Discriminant Coefficient
Age: ≤30 years	1	0.766
>30 years	2	
Referral status: Nonreferred	1	1.581
Referred	2	
ANC attendance: attended	1	1.166
Not attended	2	
HIV serostatus: negative	1	0.988
Positive	2	
Type of delivery: not surgical delivery	1	0.614
Surgical delivery	2	

Constant = −6.344.

## Data Availability

The summarised data used to support this study have been included in this manuscript. The datasets generated and analysed during the current study are not publicly available and remain as the property of Lacor Hospital but are available from the corresponding author on reasonable request.

## Abbreviations

(LMICs): Low and middle-income countries,
(SSA): Sub-Saharan Africa,
(MMR): Maternal mortality ratio,
(SDG): Sustainable Development Goals,
(WHO): World Health Organization,
(MoH): Ministry of Health
(MDR): Maternal death review,
(MCH): Maternal child health,
(ANC): Antenatal care,
(IQR): Inter quartile range,
(C/S): Caesarean section.
